# The Pulmonic Valve in Pulmonitis

**Published:** 1916-07

**Authors:** Arch Elkin

**Affiliations:** Atlanta, Ga.


### Journal-Record of Medicine

Successor to Atlanta Medical and Surgical Journal, Established 1855
and Southern Medical Record, Established 1870

**OWNED BY THE ATLANTA MEDICAL JOURNAL COMPANY**

PUBLISHED MONTHLY

*Official Organ Fulton County Medical Society, State Examing Board, Atlanta, Birmingham and Atlantic Railroad Surgeon's Association, Chattahoochee Valley Medical and Surgical Association, Etc.*

RICHARD R. DALY, M. D., Editor

A. W. STIRLING, M. D., C. M., D. P. H.; J. S. HURT, B. Ph., M.D.
GEO. M. NILES, M. D., W. J. LOVE, M. D., (Ala.); Associate Editors

E. W. ALLEN, Business Manager

**COLLABORATORS**

W. F. WESTMORELAND, M. D., General Surgery
F. W. McRAE, M. D., Abdominal Surgery
ALLEN H. BUNCE, Medical Societies
E. B. BLOCK, M. D., Diseases of the Nervous System
MICHAEL HOKE, M. D., Orthopedic Surgery
CYRUS W. STRICKLER, M. D., Legal Medicine and Medical Legislation
E. C. DAVIS, A. B., M. D., Obstetrics
E. G. JONES, A. B., M. D., Gynecology
ALLEN H. BUNCE, M. D., Pathology and Bacteriology
R. T. DORSEY, Jr., B. S., M. D., Medicine
L. M. GAINES, A. B., M. D., Internal Medicine
GEO. C. MIZELL, M. D., Diseases of the Stomach and Intestines
L. B. CLARKE, M. D., Pediatrics
EDGAR PAULLIN, M. D., Opsonic Medicine
THEODORE TOEPEL, M. D., Mechano Therapy
R. R. DALY, M. D., Diseases of the Eye, Ear, Nose and Throat
E. G. BALLENGER, M. D., Diseases of the Genito-Urinary Organs

VOL. LXIII. ATLANTA, GA., JULY, 1916. NO. 4

### THE PULMONIC VALVE IN PULMONITIS.

BY ARCH ELKIN, M.D., ATLANTA, GA.

On account of its intimate association with the lungs, and because of its mechanical integrity, the heart often proves a gate-way for pulmonary study, and in some cases is an open sesame to information hidden beneath the pleuritic excursions.

Nothing in medical diagnosis is probably more interesting than the affect of one diseased organ upon another; nor is anything more important in the prognostication of disease than the recognition of dangerous a-priori investigation.

The reasoning of "cause to effect" rather than from "effect back to cause," is an obvious barrier to a correct interpretation of conditions wherever found, and is a fault of which we are all too often guilty. The spirit of assumption, in other words, from hackneyed and time-honored expressions concerning sequelae, is apt to become too prominent in the everyday work of the diagnostician.

The value, then, of studying the heart with its variable sounds, in connection with the lungs, depends entirely upon the reasoning of the examiner, and the differentiation of primary change and secondary or indirect variation, which in the finer points of physical examination is a confusing situation. The close association of secondary heart change with cardiac compensation with the original pathology confined within the pericardium has led us to consider and study sound variation as secondary to primary heart disease, eliminating the mechanics of the pulmonary system and the effect a lung disturbance may have upon the heart sounds.

Heart sounds, normal or abnormal, are but the result of mechanical valve activity, and are transmitted directly, and almost from their exact anatomical site, through the chest wall. The variation of the different sounds in the several situations we have learned to differentiate because of their duration, intensity and pitch and because of constant comparison of the normal and abnormal we are able to recognize a deviation from the rhythm of correct valve closure.

The lungs, through direct transmission of sound are able to affect the sound variation in the heart just as one valve may affect another and the reason is just as mechanical. The semilunar valves of the pulmonary artery afford the same guard, so far as sound, at least, is concerned, as does the mitral in the left cycle, and are probably more susceptible to change from minor influences than are any of the intra-cardiac apertures. The reason for this is probably because the origin of the trouble is from behind the valve and in the direction of the blood current, whereas with the secondary mitral change, for instance, we usually have the trouble from in front of the valve, as in aortic insufficiency. So that the minor influence that would probably give no sound variation in the mitral region would produce perceptible change in the pulmonary semilunar valves.

The effect upon the pulmonary sounds is usually that of accentuation, and this occurring in the absence of any other heart lesion would lead us to the suspicion that the trouble came from the pulmonary side of the valve, and that the cause was probably to be found in the lungs. The usual even murmur heard over the pulmonary valve in the normal heart is maintained by a certain pulmonary pressure together with perfect valve closure and any cause that affects either, naturally disturbs the note produced, so that any conditon in the lungs that may change pulmonary pressure results directly on the valve sounds.

In any form of pulmonitis, whether in the acute consolidations or with the progressive formation of fibroid tissue, the intra-pulmonary pressure is increased. This is of value particularly from a diagnostic standpoint in those cases in which there is the slow progressive change, as in phthisis. The text books, while mentioning the phenomena above stated, treat it lightly by merely paying their respects in passing. Most of them seem to place little importance upon it as a diagnostic aid. Personally, I regard it of great importance and in suspected cases of early pulmonary tuberculosis, I search as diligently for the valve disturbance as I do for the real lesion.

Proof of its value is given in the haemorrhage cases of tuberculosis. Under constant supervision, the haemorrhage can sometimes be forecasted by the accentuation of the pulmonic sound and often can be avoided by the proper precautionary measures.

The interpretation, then, of the accentuated pulmonic sound, coupled with the reasoning of the examiner, leads us into the study of "effect to cause," supplanting that of "cause to effect," and it is to this pitfall that I desire to particularly call your attention.

In studying secondary change upon an adjacent organ, we are likely to accept pathological results by comparison, particularly when the histclogical picture is identical. The index to conclusons being the assumption that symptomatology does not vary when histology and pathology are in accord. A striking illustration of this is shown in the comparison of plastic pleurisy and pericarditis. In the former we always have pain with each excursion of the pleura, bringing into contact the roughened surfaces. In the latter, with the histology and pathology practically identical, we often see cases where pain is either entirely absent or infrequent. Some of the textbooks even give as a cardinal symptom of pericarditis—pain. It is possible that the assumption was made by comparison.

So, in the study of the pulmonic variation, if we presume the course of tracing the effect back to the cause, we may often find the primary lesion, deep-seated, in the pulmonary apparatus.

Suite 907 Candler Building.

